# Supplementary material for: α7 nicotinic acetylcholine receptors regulate radial glia fate in the developing human cortex
Source: Nat Commun. 2025 Jul 1;16:5925. doi: 10.1038/s41467-025-61167-5 (PMC12217762; doi:10.1038/s41467-025-61167-5)
Supplement: Supplementary file 1 — Supplementary Information [file 41467_2025_61167_MOESM1_ESM.pdf]

# Supplementary Data

***α7 nicotinic acetylcholine receptors regulate radial glia fate in the developing human cortex***

Tanzila Mukhtar[1,2,#,*], Clara-Vita Siebert[1,2,3,*], Yuejun Wang[1,2,*], Mark-Phillip Pebworth[1,2,4,*], Matthew L. White[1,2], Tianzhi Wu[1,2], Tan Ieng Huang[1,2], Guolong Zuo[1,2], Jayden Ross[1,2], Jennifer Baltazar[1,2], Varun Upadhyay[1,2], Merut Shankar[1,2], Li Zhou[1,2], Isabel Lombardi-Coronel[1,2], Ishaan Mandala[1,2], Manal A. Adam[1,2], Shaohui Wang[1,2], Qiuli Bi[1,2], Marco F.M. Hoekman[3], Jingjing Li[1,2] and Arnold R. Kriegstein[1,2,#]

1. Department of Neurology, University of California, San Francisco (UCSF), San Francisco, CA, USA, 94143
2. The Eli and Edythe Broad Center of Regeneration Medicine and Stem Cell Research, UCSF, San Francisco, CA, USA, 94143
3. Swammerdam Institute for Life Sciences, University of Amsterdam, 1098 XH Amsterdam, The Netherlands
4. Allen Institute of Immunology, Seattle, WA, USA, 98109

*These authors contributed equally

#Correspondence should be addressed to Tanzila.mukhtar@ucsf.edu and Arnold.Kriegstein@ucsf.edu

## Supplementary methods

*Lentiviral Knockdown*

Multiple small hairpin-RNAs (shRNAs) directed against *CHRNA7* and *CHRFAM7A* were designed (see table 4), and lentivirus generated and packaged in 293T cells. Supernatant was collected after 48 and 72h and ultracentrifuged. To test the knockdown efficiency using RT-qPCR, dissociated primary cortices (GW14-18) of three biological replicates was infected with lentiviruses carrying shRNAs, shCHRNA7 and shCHRFAM7A on day 0 and 3. shScrambled (shCONTROL) was used as a negative control for the experiment. RNA was harvested for RT-qPCR on day 7.

**Table 4.** shRNA sequences

| Target gene | Forward | ID |
|---|---|---|
| *shCONTROL (pLKO-RFP)* | CAACAAGATGAAGAGCACCAA | 69040 (Addgene) |
| *shCHRNA7_1* | CCGGGCAAATGTCTTGGACAGATCACTCGAGTGATCTG TCCAAGACATTTGCTTTTTTG | TRCN0000434356 (Sigma ID) |
| *shCHRNA7_2* | CCGGGATCACTATTTACAGTGGAATCTCGAGATTCCAC TGTAAATAGTGATCTTTTTG | TRCN0000061169 (Sigma ID) |
| *shCHRNA7_3* | CCGGCGAGTTCCAGAGGAAGCTTTACTCGAGTAAAGCT TCCTCTGGAACTCGTTTTTG | TRCN0000061170 (Sigma ID) |
| *shCHRFAM7A_1* | CCGGCAGAACCCATAGGACAAATAACTCGAGTTATTTG TCCTATGGGTTCTGTTTTTG | TRCN0000241872 (Sigma ID) |
| *shCHRFAM7A_2* | CCGGGGCAGATATCAGTGGCTATATCTCGAGATATAGC CACTGATATCTGCCTTTTTG | TRCN0000241869 (Sigma ID) |

| | | |
|---|---|---|
| *shCHRFAM7A_3* | CCGGGCAAACTGCGATATTGCTGATCTCGAGATCAGCA ATATCGCAGTTTGCTTTTTG | TRCN0000060468 (Sigma ID) |

*RNA isolation and qPCR validation of candidate genes*

RNA was extracted from primary cortical tissue using the QIAGEN RNeasy Plus Micro Kit following the manufacturer's recommendation. RNA concentrations were quantified using a NanoDrop. cDNA was prepared using the SuperScript™ IV VILO™ Master Mix with ezDNase™ kit (Invitrogen; #11766050) and qPCRs were performed with LightCycler® 480 SYBR Green I Master Kit (Roche; #4707516001). Primers were designed and purchased from IDT and primer sequences for target genes are shown in table 5.

**Table 5.** Primer sequences of target genes.

| **Target gene** | **Forward** | **Reverse** |
|---|---|---|
| GAPDH | GGAGCGAGATCCCTCCAAAAT | GGCTGTTGTCATACTTCTCATGG |
| B-ACTIN | CATGTACGTTGCTATCCAGGC | CTCCTTAATGTCACGCACGAT |
| CHRNA7 | GCTGGTCAAGAACTACAATCCC | CTCATCCACGTCCATGATCTG |
| CHRFAM7A | TGCATCTACCAGCATTTTCAGT | GCAATATCGCAGTTTGCAGCTAT |
| CACNA1A | TGGCACTCTACAACCCCATC | TCGGTGATCTTTTTGGCGTATT |
| BMP2 | ACTACCAGAAACGAGTGGGAA | GCATCTGTTCTCGGAAAACCT |
| UNC5C | ACCTGTACTGTAAAGCAAGCC | GGACAATGAGACCGGAAGTTT |
| GABRG3 | GTGGGTCTTGGCTCCAAAATC | ACGTCAATTACGGTCGGTTTTA |
| TKTL1 | ACAAGCAGTCAGATCCAGAGA | TAGCTGGCCCTGTCGAAGTA |
| CXCL10 | GTGGCATTCAAGGAGTACCTC | TGATGGCCTTCGATTCTGGATT |
| HTR2A | CTTTGTGCAGTCTGGATTTACCT | ACTGATATGGTCCAAACAGCAAT |

## Supplementary Figures

**a**

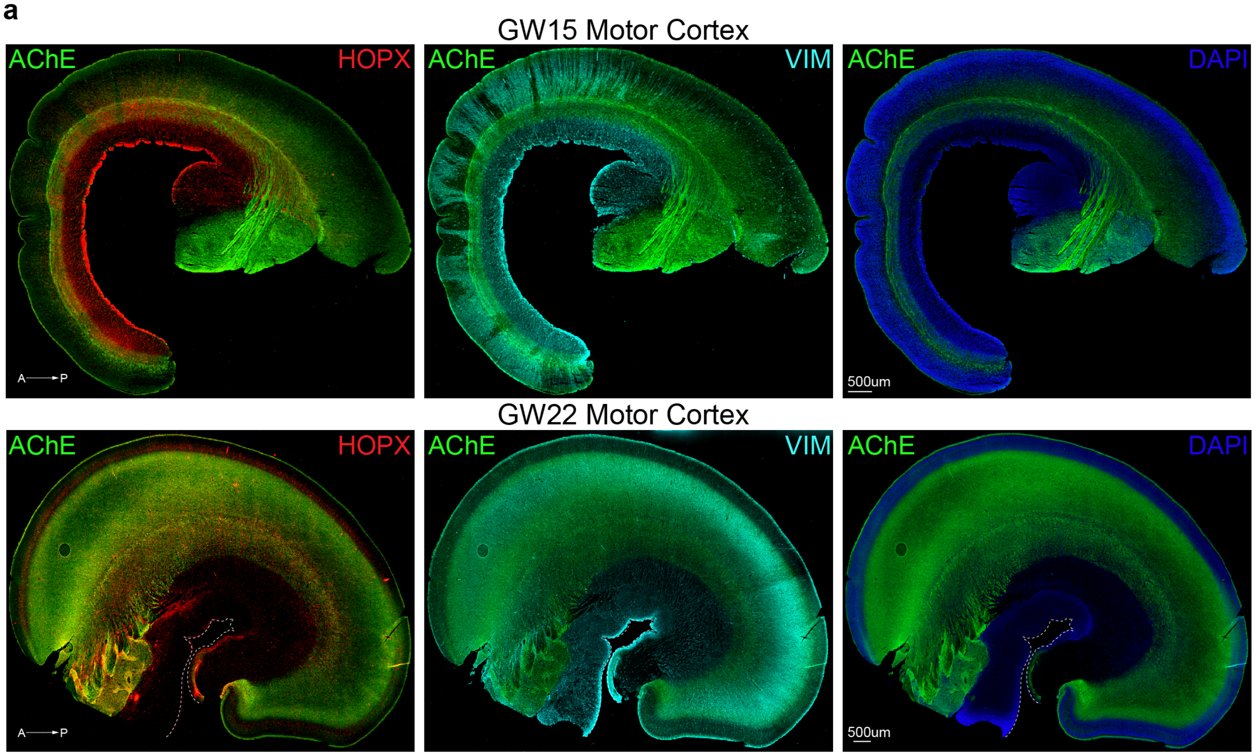


**b**

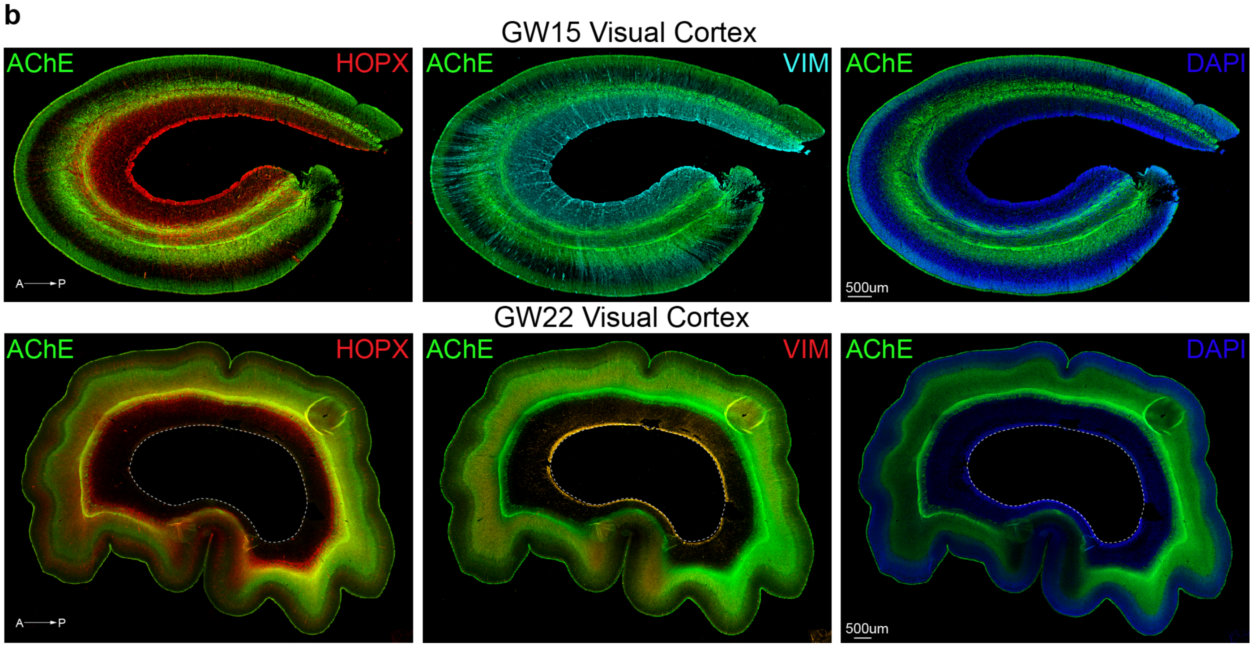

**Supplementary Fig. 1. AChE is expressed in cortical areas of the developing human brain.** **a.** Representative images showing immunostaining of AChE+ cholinergic fibers, HOPX+ oRG and VIM+ RG fibers at GW15 and GW22 in motor cortex. Scale bar = 500um. **b.** Representative images showing immunostaining of AChE+ cholinergic fibers, HOPX+ oRG and VIM+ RG fibers at GW15 and GW22 in visual cortex. Scale bar = 500um. Abbreviations, A-P : anterior-posterior.

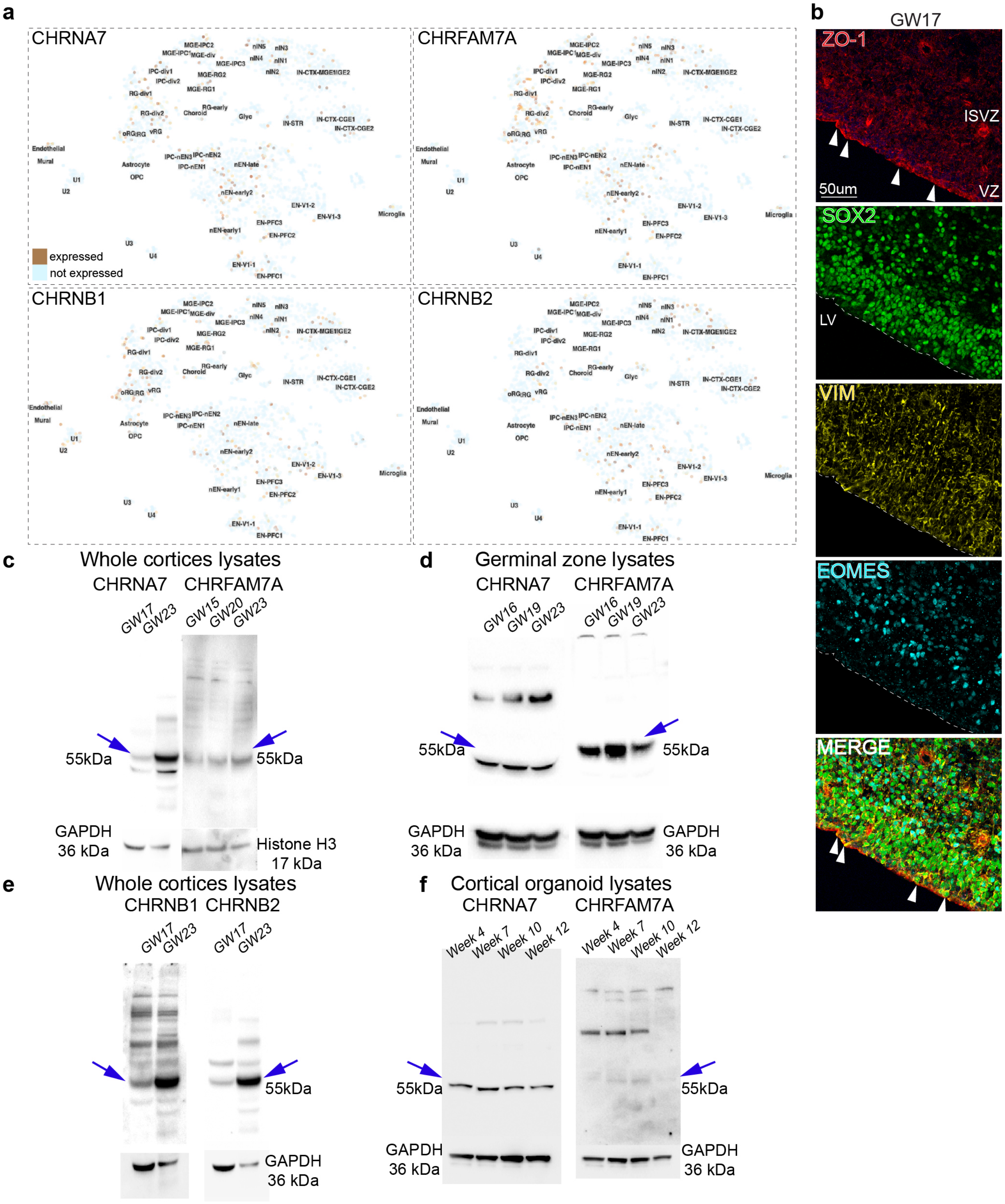
a
CHRNA7
CHRFAM7A
CHRNB1
CHRNB2
expressed
not expressed
b
GW17
ZO-1
ISVZ
VZ
50um
SOX2
LV
VIM
EOMES
MERGE
c
Whole cortices lysates
CHRNA7
CHRFAM7A
GW17
GW23
GW15
GW20
GW23
55kDa
55kDa
GAPDH
36 kDa
Histone H3
17 kDa
d
Germinal zone lysates
CHRNA7
CHRFAM7A
GW16
GW19
GW23
GW16
GW19
GW23
55kDa
55kDa
GAPDH
36 kDa
GAPDH
36 kDa
e
Whole cortices lysates
CHRNB1
CHRNB2
GW17
GW23
GW17
GW23
55kDa
GAPDH
36 kDa
f
Cortical organoid lysates
CHRNA7
CHRFAM7A
Week 4
Week 7
Week 10
Week 12
Week 4
Week 7
Week 10
Week 12
55kDa
55kDa
GAPDH
36 kDa
GAPDH
36 kDa

**Supplementary Fig. 2. Expression of nAChR receptor subunits across developing human cortex. a.** *In-silico* analysis of published single-cell sequencing data[2] shows RNA expression of nAChR subunits CHRNA7, CHRFAM7A, CHRNB1 and CHRNB2 in the developing human cortex, including in progenitor cells. **b.** Co-immunostaining of tight junction protein 1 (ZO-1) with SOX2, VIM and EOMES showing expression in RG endfeet along the apical border of the VZ (ZO-1 staining shown by arrowheads). **c.** Western blotting confirms protein expression of CHRNA7 and CHRFAM7A across neurogenic and gliogenic time points during second trimester (n=2). **d.** Western blotting performed with protein samples enriched for germinal zone from GW16-19 shows high expression of CHRNA7 and CHRFAM7A. **e**. Western blotting for alternate nAChRs, CHRNB1 and CHRNB2, from cortical samples shows high expression of receptor subunits at GW17 and GW23. **f**. Western blotting of cortical organoids shows CHRNA7 and CHRFAM7A expression across weeks 4-12. Abbreviations : VZ : ventricular zone, iSVZ : inner subventricular zone, LV : lateral ventricle.

NAChR subunit CHRNA7 is expressed in Germinal zones

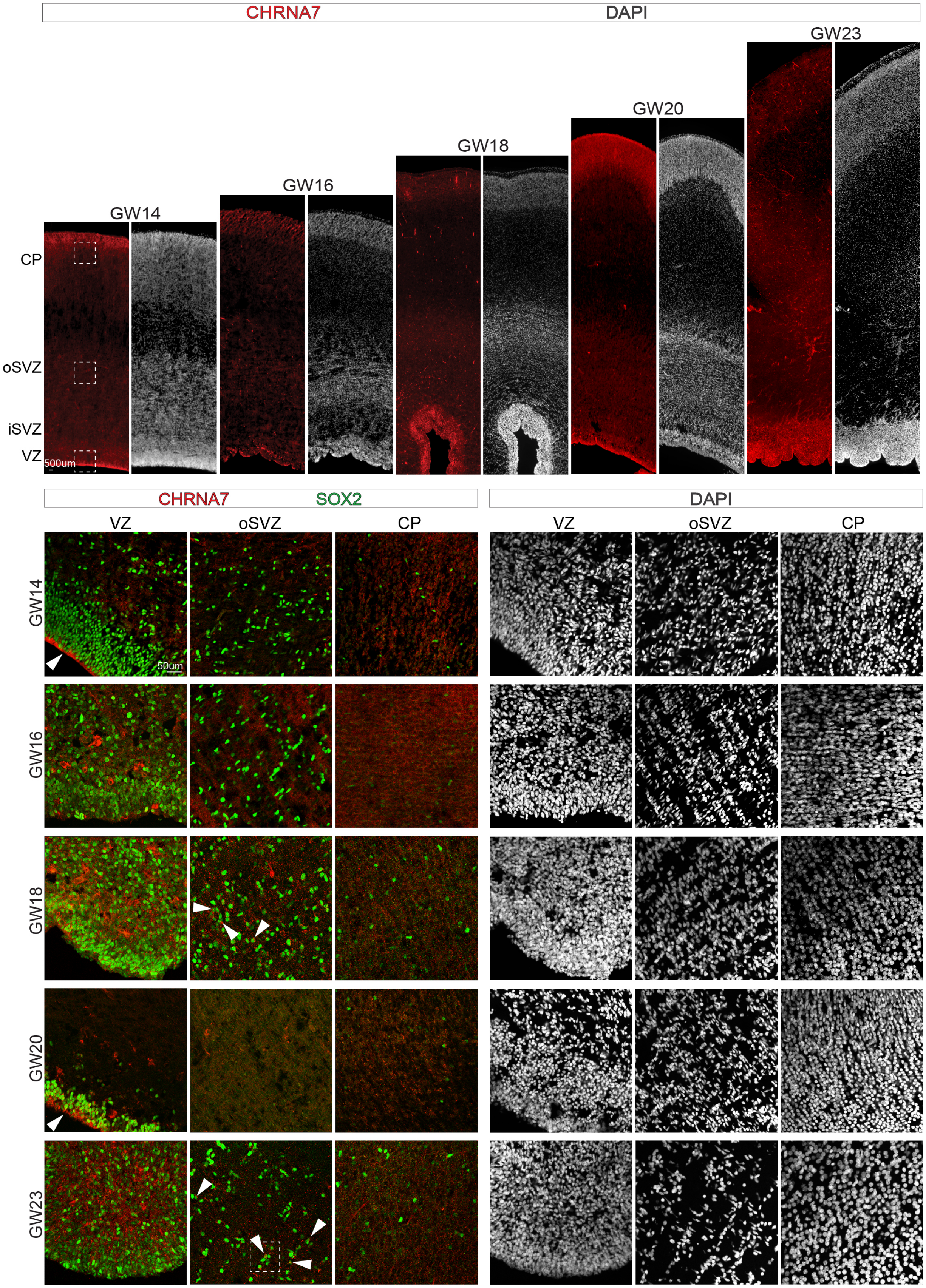

**Supplementary Fig. 3. NAChR subunit CHRNA7 is expressed in germinal zones across human cortical development.** Representative images from VZ to CP show co-immunostaining for CHRNA7 (nAChR), SOX2 (radial glia, outer radial glia), and DAPI (nuclei) in cortical sections from GW14, GW16, GW18, GW20, and GW23, scale bar = 500um. Magnified images highlight the expression of CHRNA7 protein (arrowheads) in the VZ, oSVZ, and CP throughout development, scale bar = 50um. VZ- Ventricular zone, iSVZ- Inner subventricular zone OSVZ- Outer subventricular zone, CP- Cortical plate.

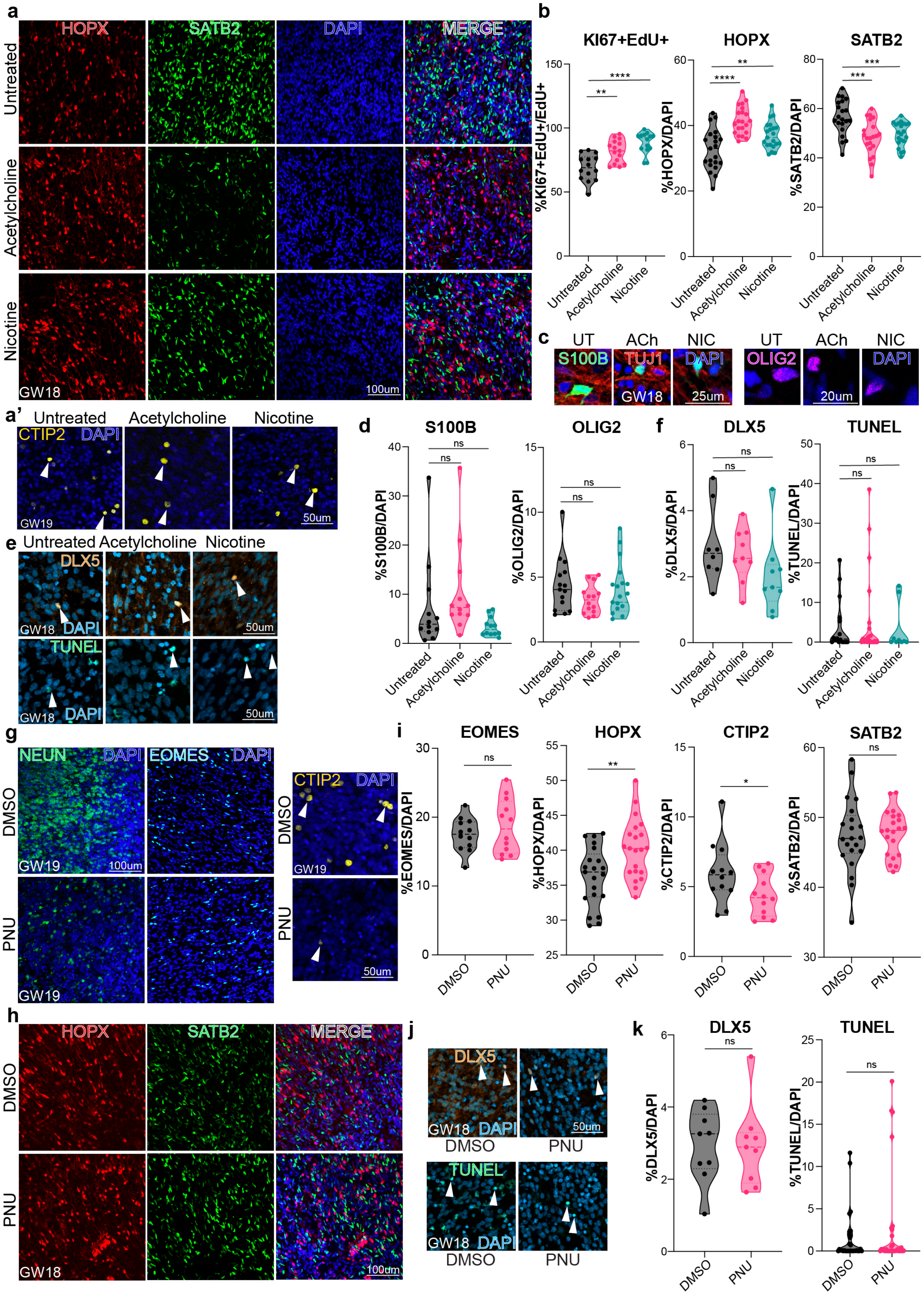

a
HOPX
SATB2
DAPI
MERGE
Untreated
Acetylcholine
Nicotine
GW18
100um
b
KI67+EdU+
HOPX
SATB2
%KI67+EdU+/EdU+
%HOPX/DAPI
%SATB2/DAPI
Untreated
Acetylcholine
Nicotine
c
UT
ACh
NIC
S100B
TUJ1
DAPI
OLIG2
GW18
25um
20um
a'
CTIP2
DAPI
GW19
50um
d
S100B
OLIG2
%S100B/DAPI
%OLIG2/DAPI
ns
f
DLX5
TUNEL
%DLX5/DAPI
%TUNEL/DAPI
e
GW18
g
NEUN
EOMES
DMSO
PNU
i
EOMES
CTIP2
%EOMES/DAPI
%CTIP2/DAPI
h
j
k

**Supplementary Fig. 4. Activation of cholinergic signaling in primary cortical organotypic slices by agonist treatment increases oRGs and inhibits neuronal differentiation**. **a.** Representative images showing immunostained HOPX+, SATB2+ cells co-stained with DAPI in GW18 organotypic slices, post agonist treatment. Scale bar = 100um. **a'.** Representative images of GW19 organotypic slices showing immunostained CTIP2+ cells co-stained with DAPI in the IZ post agonist treatment. Scale bar = 50um. **b.** Quantification of HOPX and SATB2 over total DAPI, post agonist treatment (n=3). We observed a significant increase in HOPX+ oRG cells with 41.5% in ACh (p=1.97E-07) and 37% (p=0.004) in nicotine, compared to 32% in control. **c.** Representative images of GW18 organotypic slices showing immunostained S100B+ and OLIG2+ cells co-stained with DAPI in the oSVZ post agonist treatments. Scale bar = 25um, 20um. **d.** Quantification of S100B and OLIG2 over total DAPI, post agonist treatment (n=3) shows no significant changes. **e.** Representative images showing immunostained DLX5+ cells and TUNEL staining in GW18 organotypic slices post agonist treatment. Scale bar = 50um. **f.** Quantification of DLX5 and TUNEL over total DAPI post agonist treatment (n=3). We observed no significant changes in DLX5 interneurons or cell death across conditions**. g,h.** Representative images showing NEUN+, EOMES+, CTIP2+, HOPX+, and SATB2+ immunostaining in GW18 and 19 organotypic slices post specific agonist treatment and DMSO control. Scale bar = 100um, and 50um for CTIP2. **i.** Quantification of cell type markers, EOMES, HOPX, CTIP2 and SATB2 in PNU-treated slice cultures compared to DMSO control. PNU did not change EOMES+ IPC numbers. HOPX+ cells increased to 40.2% (p=0.003) from 36.2% in control. We observed a reduction in neurons, impacting CTIP2+ deep layer neurons, with no change in upper layer SATB2+ neurons. **j.** Immunostaining for DLX5+ cells and TUNEL in GW18 organotypic slices post specific agonist treatment. Scale bar = 50um. **k.** Quantification for DLX5 and TUNEL over total DAPI, post specific agonist treatment showed no change (n=3), (Source Data), two-sided t-test, *p=0.05, **p=0.01, ***p=0.001, ****p=0.0001. ns= not significant.

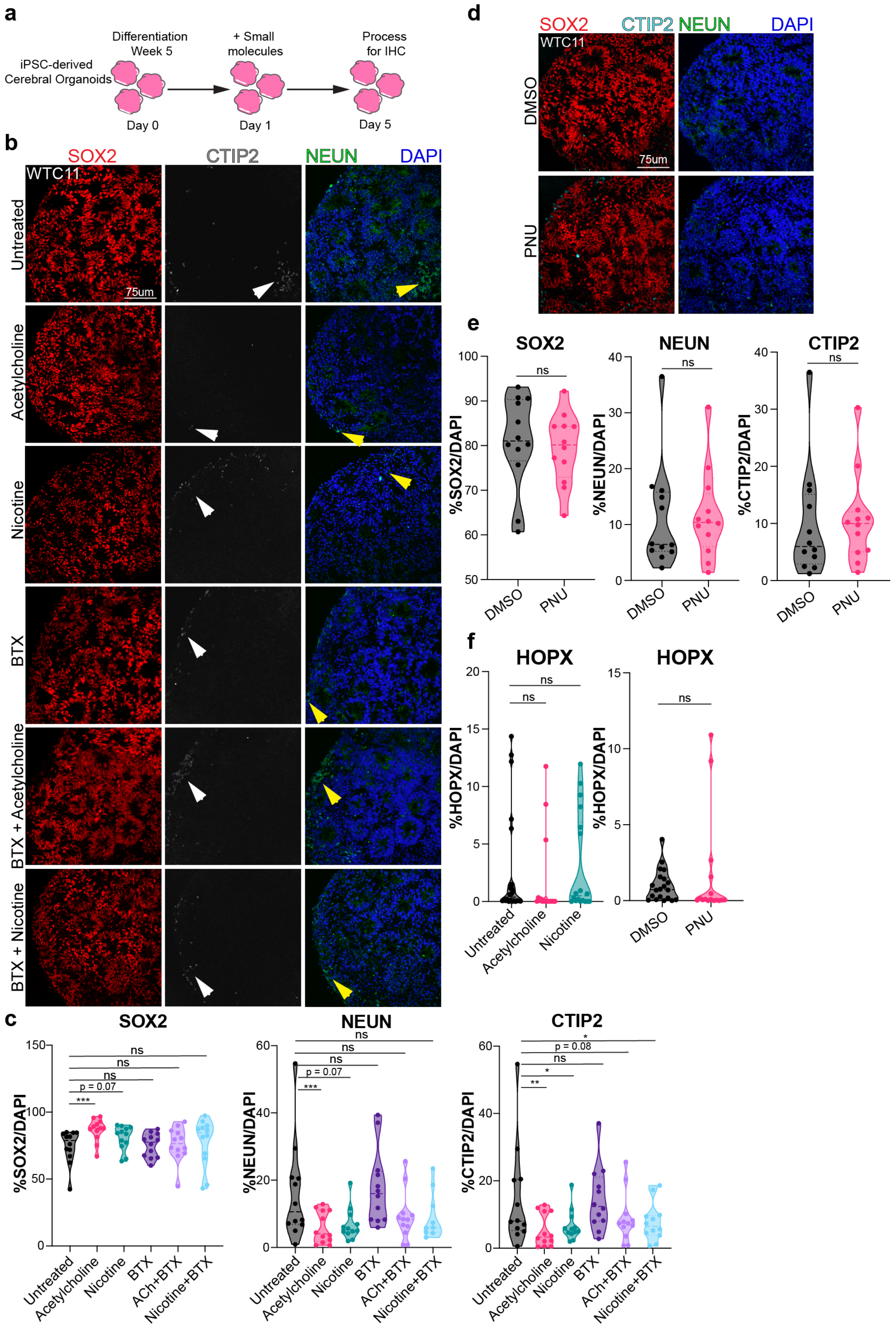
a
iPSC-derived Cerebral Organoids
Differentiation Week 5
+ Small molecules
Process for IHC
Day 0
Day 1
Day 5
b
SOX2
CTIP2
NEUN
DAPI
WTC11
Untreated
Acetylcholine
Nicotine
BTX
BTX + Acetylcholine
BTX + Nicotine
75um
c
SOX2
NEUN
CTIP2
%SOX2/DAPI
%NEUN/DAPI
%CTIP2/DAPI
ns
p = 0.07
p = 0.08
***
**
*
Untreated
Acetylcholine
Nicotine
BTX
ACh+BTX
Nicotine+BTX
d
SOX2
CTIP2
NEUN
DAPI
WTC11
DMSO
PNU
75um
e
SOX2
NEUN
CTIP2
%SOX2/DAPI
%NEUN/DAPI
%CTIP2/DAPI
ns
DMSO
PNU
f
HOPX
%HOPX/DAPI
ns
Untreated
Acetylcholine
Nicotine
DMSO
PNU

**Supplementary Fig. 5. Activation of cholinergic signaling in early cortical organoids shows comparable trends to primary tissue. a.** Scheme of treatment in cortical organoids with relatively broad (ACh) and specific (nicotine, PNU) agonists, the antagonist, a-bungarotoxin (BTX), and combinations of agonist + antagonist (ACh+BTX and nicotine +BTX). Three control cortical organoid lines were treated at differentiation Week 5 for five consecutive days, then PFA fixed and processed for IHC. **b.** Representative images of cortical organoids immunostained with SOX2, CTIP2 (white arrows), NEUN (yellow arrows) and DAPI. Scale bar = 75um. **c.** Quantification for SOX2, NEUN, and CTIP2 shows changes in cell types across conditions. SOX2+ progenitors increase to 86.50% (p=0.0007) with ACh and 82.31% (p=0.069) with nicotine as compared to 73.50% in control. ACh reduced NEUN+ neurons to 6% (p=0.002), and nicotine reduced them to 6.8% (0.005) from 16% in control. Similarly, CTIP2+ deep-layer neurons were reduced to 4.6% (p=0.005) and 5.6% (p=0.017) in ACh and nicotine, respectively, compared to 11.1% in control. In combination with the agonists (ACh or nicotine), the antagonist, BTX, was able to normalize the percentages of SOX2+, NEUN+ and CTIP2+ cells. BTX + ACh increased SOX2+ cells to 76.1%, while BTX+NIC increased SOX2+ cells to 77.45%, compared to 73.5% in control. We observed significant changes in NEUN+ cells, with 9.1% in BTX (p=0.03) + ACh and 8.5% in BTX + NIC (p=0.02), compared to 16.5% in UT controls. CTIP2+ cells changed slightly, with 8.8% in BTX + ACh (p=0.08) and 7.7% in BTX + nicotine (p=0.03), compared to the control with 11.1% cells. **d.** Representative images of WTC-11 cortical organoids stained for SOX2, NEUN, and DAPI, post treatment with specific agonist and DMSO control. Scale bar = 75um **e.** Quantification of SOX2 and NEUN post PNU treatment compared to DMSO control. **f.** Quantifications for HOPX+ oRG post agonist treatment show no changes (n=3) with details specified in the Source Data, two-sided test, *p=0.05, **p=0.01, ***p=0.001, ****p=0.0001. ns= not significant.

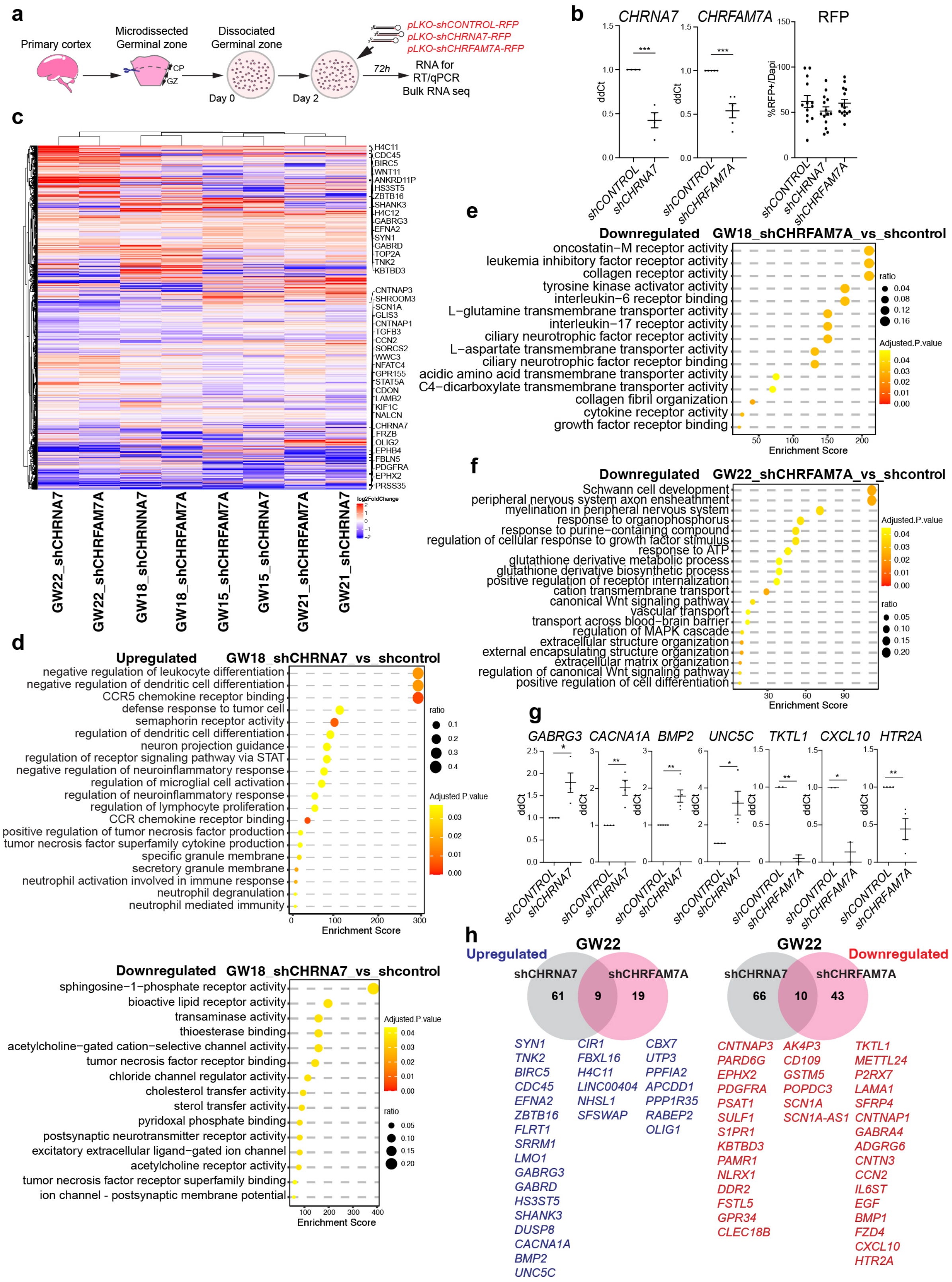
a
Primary cortex
Microdissected Germinal zone
Dissociated Germinal zone
CP
GZ
Day 0
Day 2
pLKO-shCONTROL-RFP
pLKO-shCHRNA7-RFP
pLKO-shCHRFAM7A-RFP
72h
RNA for RT/qPCR Bulk RNA seq
b
CHRNA7
CHRFAM7A
RFP
ddCt
%RFP+/Dapi
shCONTROL
shCHRNA7
shCHRFAM7A
c
H4C11
CDC45
BIRC5
WNT11
ANKRD11P
HS3ST5
ZBTB16
SHANK3
H4C12
GABRG3
EFNA2
SYN1
GABRD
TOP2A
TNK2
KBTBD3
CNTNAP3
SHROOM3
SCN1A
GLIS3
CNTNAP1
TGFB3
CCN2
SORCS2
WWC3
NFATC4
GPR155
STAT5A
CDON
LAMB2
KIF1C
NALCN
CHRNA7
FRZB
OLIG2
EPHB4
FBLN5
PDGFRA
EPHX2
PRSS35
log2FoldChange
GW22_shCHRNA7
GW22_shCHRFAM7A
GW18_shCHRNA7
GW18_shCHRFAM7A
GW15_shCHRFAM7A
GW15_shCHRNA7
GW21_shCHRFAM7A
GW21_shCHRNA7
d
Upregulated GW18_shCHRNA7_vs_shcontrol
negative regulation of leukocyte differentiation
negative regulation of dendritic cell differentiation
CCR5 chemokine receptor binding
defense response to tumor cell
semaphorin receptor activity
regulation of dendritic cell differentiation
neuron projection guidance
regulation of receptor signaling pathway via STAT
negative regulation of neuroinflammatory response
regulation of microglial cell activation
regulation of neuroinflammatory response
regulation of lymphocyte proliferation
CCR chemokine receptor binding
positive regulation of tumor necrosis factor production
tumor necrosis factor superfamily cytokine production
specific granule membrane
secretory granule membrane
neutrophil activation involved in immune response
neutrophil degranulation
neutrophil mediated immunity
ratio
Adjusted.P.value
Enrichment Score
Downregulated GW18_shCHRNA7_vs_shcontrol
sphingosine−1−phosphate receptor activity
bioactive lipid receptor activity
transaminase activity
thioesterase binding
acetylcholine−gated cation−selective channel activity
tumor necrosis factor receptor binding
chloride channel regulator activity
cholesterol transfer activity
sterol transfer activity
pyridoxal phosphate binding
postsynaptic neurotransmitter receptor activity
excitatory extracellular ligand−gated ion channel
acetylcholine receptor activity
tumor necrosis factor receptor superfamily binding
ion channel - postsynaptic membrane potential
e
Downregulated GW18_shCHRFAM7A_vs_shcontrol
oncostatin−M receptor activity
leukemia inhibitory factor receptor activity
collagen receptor activity
tyrosine kinase activator activity
interleukin−6 receptor binding
L−glutamine transmembrane transporter activity
interleukin−17 receptor activity
ciliary neurotrophic factor receptor activity
L−aspartate transmembrane transporter activity
ciliary neurotrophic factor receptor binding
acidic amino acid transmembrane transporter activity
C4−dicarboxylate transmembrane transporter activity
collagen fibril organization
cytokine receptor activity
growth factor receptor binding
f
Downregulated GW22_shCHRFAM7A_vs_shcontrol
Schwann cell development
peripheral nervous system axon ensheathment
myelination in peripheral nervous system
response to organophosphorus
response to purine−containing compound
regulation of cellular response to growth factor stimulus
response to ATP
glutathione derivative metabolic process
glutathione derivative biosynthetic process
positive regulation of receptor internalization
cation transmembrane transport
canonical Wnt signaling pathway
vascular transport
transport across blood−brain barrier
regulation of MAPK cascade
extracellular structure organization
external encapsulating structure organization
extracellular matrix organization
regulation of canonical Wnt signaling pathway
positive regulation of cell differentiation
g
GABRG3
CACNA1A
BMP2
UNC5C
TKTL1
CXCL10
HTR2A
h
Upregulated
GW22
shCHRNA7
shCHRFAM7A
61
9
19
SYN1
TNK2
BIRC5
CDC45
EFNA2
ZBTB16
FLRT1
SRRM1
LMO1
GABRG3
GABRD
HS3ST5
SHANK3
DUSP8
CACNA1A
BMP2
UNC5C
CIR1
FBXL16
H4C11
LINC00404
NHSL1
SFSWAP
CBX7
UTP3
PPFIA2
APCDD1
PPP1R35
RABEP2
OLIG1
GW22
Downregulated
shCHRNA7
shCHRFAM7A
66
10
43
CNTNAP3
PARD6G
EPHX2
PDGFRA
PSAT1
SULF1
S1PR1
KBTBD3
PAMR1
NLRX1
DDR2
FSTL5
GPR34
CLEC18B
AK4P3
CD109
GSTM5
POPDC3
SCN1A
SCN1A-AS1
TKTL1
METTL24
P2RX7
LAMA1
SFRP4
CNTNAP1
GABRA4
ADGRG6
CNTN3
CCN2
IL6ST
EGF
BMP1
FZD4
CXCL10
HTR2A

**Supplementary Fig. 6. Bulk RNA-seq reveals key molecular changes post CHRNA7 and CHRFAM7A knockdown. a.** Experimental design for bulk-RNA sequencing. Micro-dissected germinal zone (GZ) tissue was dissociated into adherent neural cultures and treated with lentiviral vectors carrying shRNA, directed against nAChRs and a Scrambled control. Cells were collected on Day 5 in culture, total RNA extracted and processed for Bulk-RNA-sequencing. RNA samples were tested for integrity using a BioAnalyzer. Only samples with a RIN score > 9.0 were sent for Bulk-RNA sequencing. **b.** RT/qPCR shows knockdown efficiency of receptor subunits after shRNA treatment. Quantifications for RFP+ cells/DAPI performed in organotypic slices, show comparable infection across conditions (n=3). **c**. DEG analyses identified candidate genes up- and downregulated upon knockdown of CHRNA7, CHRFAM7A, compared to the Scrambled control. **d.** Upregulated biological processes post CHRNA7 knockdown identified functions such as semaphorin signaling, chemokine receptor pathway, and inflammation. Downregulated molecular functions post CHRNA7 knockdown include lipid metabolism and chloride and acetylcholine channel activity. **e.** CHRFAM7A knockdown identified significant signaling pathways such as LIFR and tyrosine kinase pathways at GW18. **f**. Downregulated molecular functions following shCHRFAM7A in GW22 cultures include axon ensheathment and myelination. **g.** Additional biological replicates were collected for RT-qPCR validation of Bulk-RNA sequencing data. RT-qPCR validation for bulk-RNA-seq data using independent biological replicates recapitulated up- and down-regulation trends for candidate genes. **h.** Unique and shared targets post knockdown of CHRNA7 and CHRFAM7A identify potential downstream mechanisms mediated by these receptors. Blue- upregulated, Red- Downregulated. For RT/qPCR, N = 4-5, t-test, ***p=0.001. All DEGs and qPCR data are provided in the Source Data.

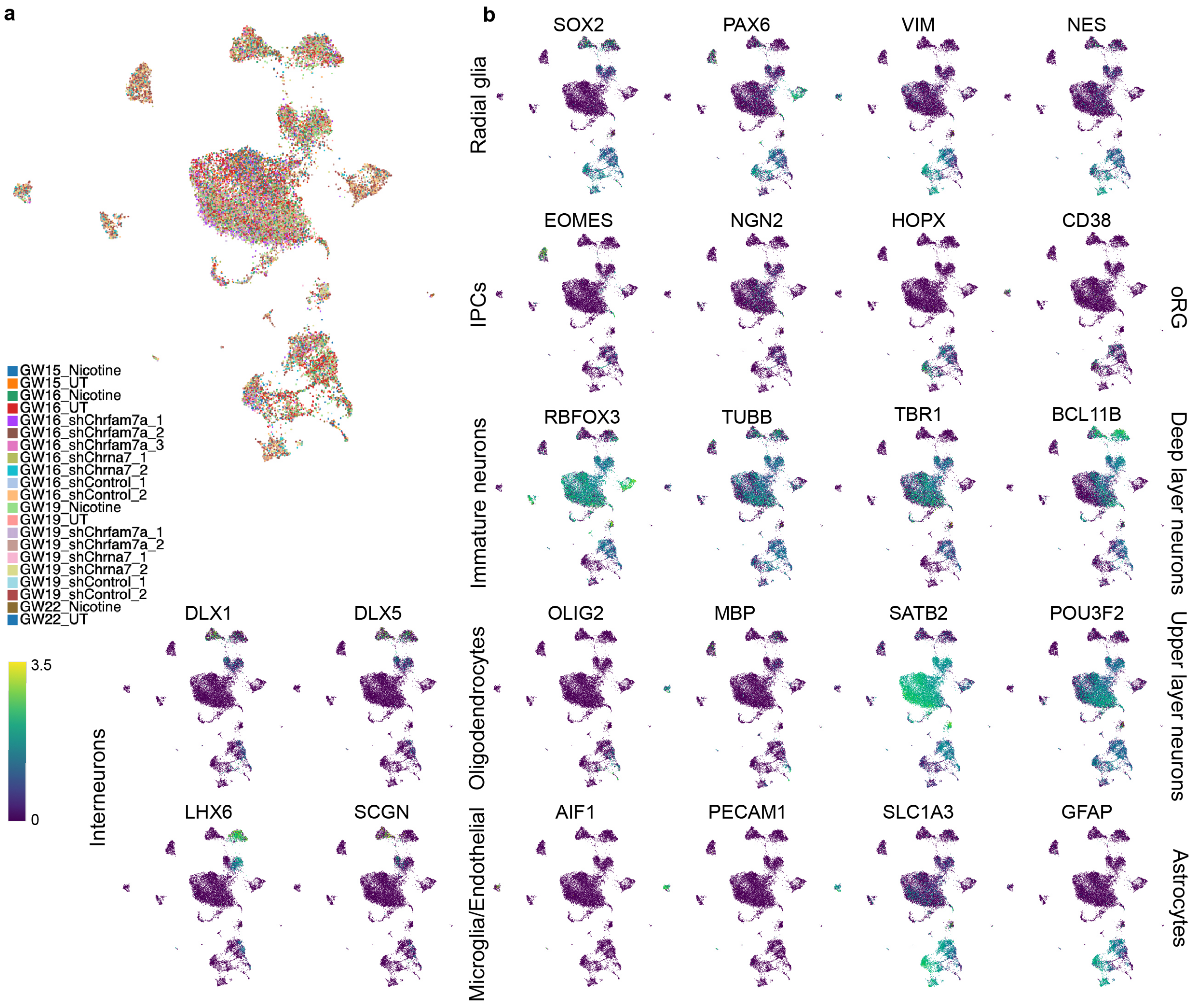


**Supplementary Fig. 7. Cell type diversity across samples post knockdown and agonist treatments. a.** Distribution of cell types across samples post knockdown and agonist treatments. **b.** Feature plots for known markers shows gene expression across diverse cell type clusters.

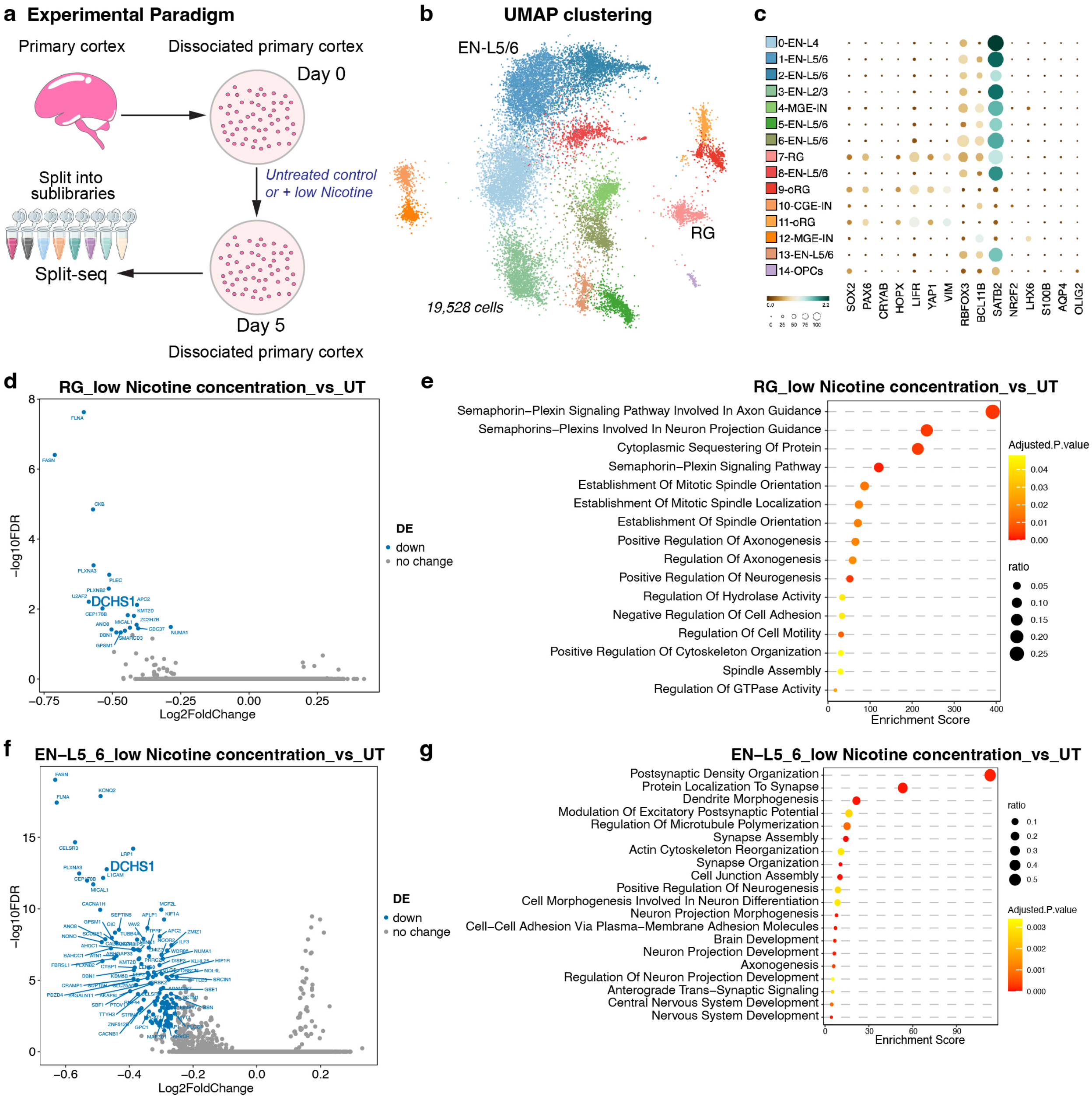
a Experimental Paradigm
Primary cortex
Dissociated primary cortex
Day 0
Untreated control
or + low Nicotine
Split into
sublibraries
Split-seq
Day 5
Dissociated primary cortex
b UMAP clustering
EN-L5/6
RG
19,528 cells
c
0-EN-L4
1-EN-L5/6
2-EN-L5/6
3-EN-L2/3
4-MGE-IN
5-EN-L5/6
6-EN-L5/6
7-RG
8-EN-L5/6
9-oRG
10-CGE-IN
11-oRG
12-MGE-IN
13-EN-L5/6
14-OPCs
SOX2
PAX6
CRYAB
HOPX
LIFR
YAP1
VIM
RBFOX3
BCL11B
SATB2
NR2F2
LHX6
S100B
AQP4
OLIG2
d RG_low Nicotine concentration_vs_UT
−log10FDR
Log2FoldChange
FLNA
FASN
CKB
DCHS1
DE
down
no change
e RG_low Nicotine concentration_vs_UT
Semaphorin−Plexin Signaling Pathway Involved In Axon Guidance
Semaphorins−Plexins Involved In Neuron Projection Guidance
Cytoplasmic Sequestering Of Protein
Semaphorin−Plexin Signaling Pathway
Establishment Of Mitotic Spindle Orientation
Establishment Of Mitotic Spindle Localization
Establishment Of Spindle Orientation
Positive Regulation Of Axonogenesis
Regulation Of Axonogenesis
Positive Regulation Of Neurogenesis
Regulation Of Hydrolase Activity
Negative Regulation Of Cell Adhesion
Regulation Of Cell Motility
Positive Regulation Of Cytoskeleton Organization
Spindle Assembly
Regulation Of GTPase Activity
Enrichment Score
Adjusted.P.value
ratio
f EN−L5_6_low Nicotine concentration_vs_UT
−log10FDR
Log2FoldChange
FASN
FLNA
KCNQ2
DCHS1
DE
down
no change
g EN−L5_6_low Nicotine concentration_vs_UT
Postsynaptic Density Organization
Protein Localization To Synapse
Dendrite Morphogenesis
Modulation Of Excitatory Postsynaptic Potential
Regulation Of Microtubule Polymerization
Synapse Assembly
Actin Cytoskeleton Reorganization
Synapse Organization
Cell Junction Assembly
Positive Regulation Of Neurogenesis
Cell Morphogenesis Involved In Neuron Differentiation
Neuron Projection Morphogenesis
Cell−Cell Adhesion Via Plasma−Membrane Adhesion Molecules
Brain Development
Neuron Projection Development
Axonogenesis
Regulation Of Neuron Projection Development
Anterograde Trans−Synaptic Signaling
Central Nervous System Development
Nervous System Development
Enrichment Score
ratio
Adjusted.P.value

**Supplementary Fig. 8. Differential gene expression with levels of Nicotine approximating fetal exposure. a.** Workflow showing nicotine exposure in primary dissociated cortical cell cultures. Cells were fixed on day 5 following the Parse Biosciences Fixation protocol and processed for Split-sequencing, a combinatorial barcoding approach where different barcodes are added in each step. Gene expression libraries were prepared and sequenced using Illumina NovaSeq2 Flow cell. **b.** UMAP clustering of single-cell data identifies expected cell types. **c.** Preliminary scRNAseq analysis reveals anticipated patterns of marker gene expression across clusters. SOX2, PAX6, CRYAB, HOPX, LIFR, YAP1, VIM are expressed in RG and oRG clusters, while RBFOX3, BCL11B and SATB2 are expressed by neuronal clusters, LHX6, NR2F2, are expressed in interneurons, S100B, AQP4 and OLIG2 in astrocytes and oligodendrocytes. **d.** Genes downregulated upon nicotine exposure in RG cells. **e.** Gene Ontology (GO) for dividing RG identifies significant pathways and processes upregulated post nicotine exposure, including Semaphorin-plexin and cell adhesion signaling. **f.** Genes downregulated upon nicotine exposure in Layer 4/5 excitatory neurons. One of the top downregulated genes is *DCHS1*, a canonical ligand of Hippo signaling. **g.** Gene Ontology (GO) for Layer 4/5 excitatory neurons identifies significant pathways and processes upregulated post nicotine exposure, including postsynaptic density organization, microtubule organization, and synapse development. All DEGs are listed in Source Data.

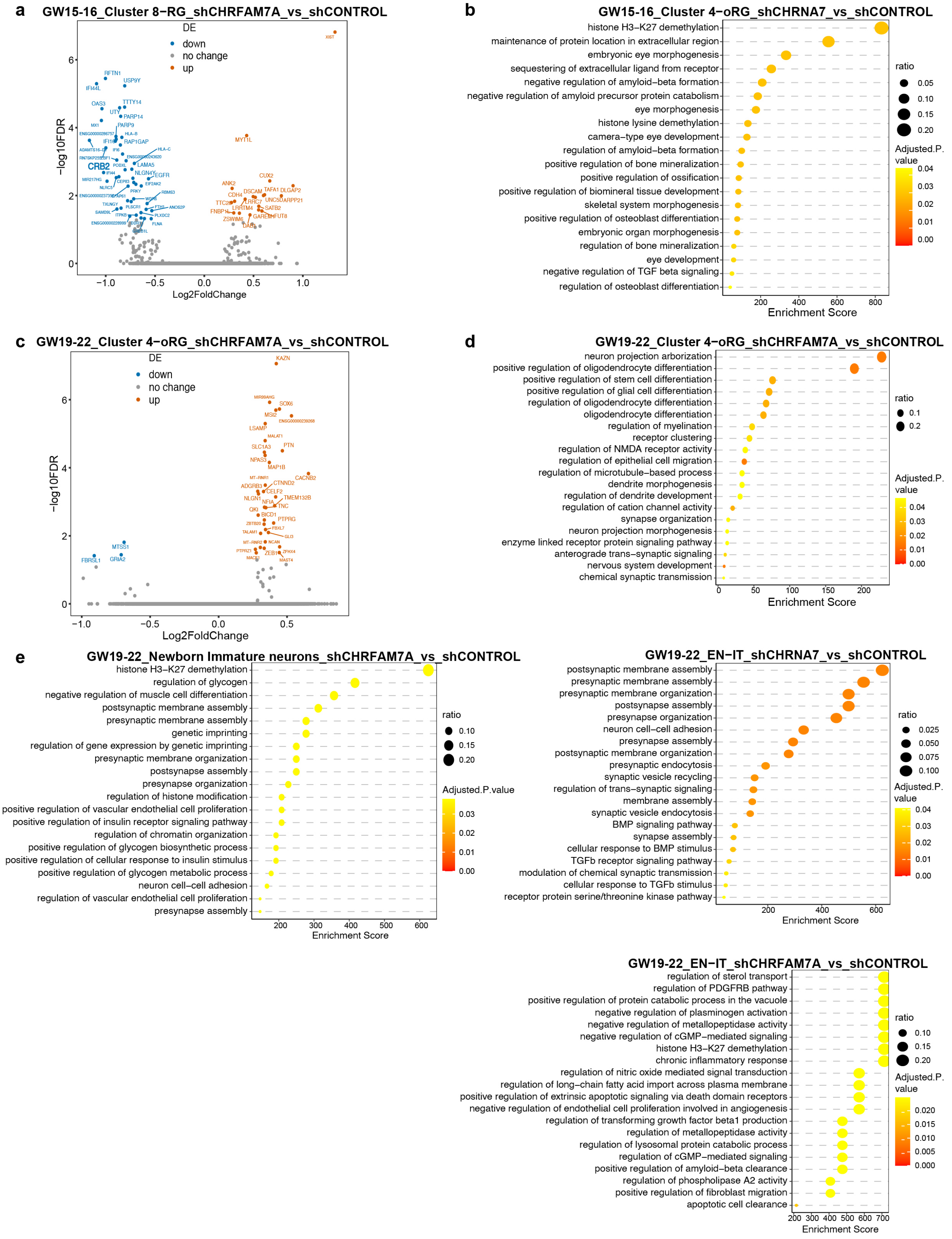
a
GW15-16_Cluster 8−RG_shCHRFAM7A_vs_shCONTROL
DE
down
no change
up
−log10FDR
Log2FoldChange
XIST
MYT1L
CRB2
b
GW15-16_Cluster 4−oRG_shCHRNA7_vs_shCONTROL
histone H3−K27 demethylation
maintenance of protein location in extracellular region
embryonic eye morphogenesis
sequestering of extracellular ligand from receptor
negative regulation of amyloid−beta formation
negative regulation of amyloid precursor protein catabolism
eye morphogenesis
histone lysine demethylation
camera−type eye development
regulation of amyloid−beta formation
positive regulation of bone mineralization
positive regulation of ossification
positive regulation of biomineral tissue development
skeletal system morphogenesis
positive regulation of osteoblast differentiation
embryonic organ morphogenesis
regulation of bone mineralization
eye development
negative regulation of TGF beta signaling
regulation of osteoblast differentiation
Enrichment Score
ratio
Adjusted.P. value
c
GW19-22_Cluster 4−oRG_shCHRFAM7A_vs_shCONTROL
DE
down
no change
up
−log10FDR
Log2FoldChange
KAZN
d
GW19-22_Cluster 4−oRG_shCHRFAM7A_vs_shCONTROL
neuron projection arborization
positive regulation of oligodendrocyte differentiation
positive regulation of stem cell differentiation
positive regulation of glial cell differentiation
regulation of oligodendrocyte differentiation
oligodendrocyte differentiation
regulation of myelination
receptor clustering
regulation of NMDA receptor activity
regulation of epithelial cell migration
regulation of microtubule−based process
dendrite morphogenesis
regulation of dendrite development
regulation of cation channel activity
synapse organization
neuron projection morphogenesis
enzyme linked receptor protein signaling pathway
anterograde trans−synaptic signaling
nervous system development
chemical synaptic transmission
Enrichment Score
ratio
Adjusted.P. value
e
GW19-22_Newborn Immature neurons_shCHRFAM7A_vs_shCONTROL
histone H3−K27 demethylation
regulation of glycogen
negative regulation of muscle cell differentiation
postsynaptic membrane assembly
presynaptic membrane assembly
genetic imprinting
regulation of gene expression by genetic imprinting
presynaptic membrane organization
postsynapse assembly
presynapse organization
regulation of histone modification
positive regulation of vascular endothelial cell proliferation
positive regulation of insulin receptor signaling pathway
regulation of chromatin organization
positive regulation of glycogen biosynthetic process
positive regulation of cellular response to insulin stimulus
positive regulation of glycogen metabolic process
neuron cell−cell adhesion
regulation of vascular endothelial cell proliferation
presynapse assembly
Enrichment Score
ratio
Adjusted.P.value
GW19-22_EN−IT_shCHRNA7_vs_shCONTROL
postsynaptic membrane assembly
presynaptic membrane assembly
presynaptic membrane organization
postsynapse assembly
presynapse organization
neuron cell−cell adhesion
presynapse assembly
postsynaptic membrane organization
presynaptic endocytosis
synaptic vesicle recycling
regulation of trans−synaptic signaling
membrane assembly
synaptic vesicle endocytosis
BMP signaling pathway
synapse assembly
cellular response to BMP stimulus
TGFb receptor signaling pathway
modulation of chemical synaptic transmission
cellular response to TGFb stimulus
receptor protein serine/threonine kinase pathway
Enrichment Score
ratio
Adjusted.P. value
GW19-22_EN−IT_shCHRFAM7A_vs_shCONTROL
regulation of sterol transport
regulation of PDGFRB pathway
positive regulation of protein catabolic process in the vacuole
negative regulation of plasminogen activation
negative regulation of metallopeptidase activity
negative regulation of cGMP−mediated signaling
histone H3−K27 demethylation
chronic inflammatory response
regulation of nitric oxide mediated signal transduction
regulation of long−chain fatty acid import across plasma membrane
positive regulation of extrinsic apoptotic signaling via death domain receptors
negative regulation of endothelial cell proliferation involved in angiogenesis
regulation of transforming growth factor beta1 production
regulation of metallopeptidase activity
regulation of lysosomal protein catabolic process
regulation of cGMP−mediated signaling
positive regulation of amyloid−beta clearance
regulation of phospholipase A2 activity
positive regulation of fibroblast migration
apoptotic cell clearance
Enrichment Score
ratio
Adjusted.P. value

**Supplementary Fig. 9. GO analysis post knockdown of nAChRs identifies key processes during gliogenic time points. a.** DEGs in RG from GW15-16 after knockdown of CHRFAM7A. We observed an upregulation of MYT1L, ANK2, DLGAP2, ARPP21, DAB1, LRRC7 and upper layer neuron markers CUX2 and SATB2, while LAMA5, PODXL, WDR6, RBMS3, RFTN1, EBF1, CRB2, EGFR were downregulated in neurogenic RG. The associated GO processes in gliogenic RG post knockdown of CHRFAM7A, include, poly-D-ribosylation, cytokine-mediated signaling and axon guidance, morphogenesis of cell epithelium, integrin signaling, and ECM structural organization (Source Data). The temporal shifts likely highlight differences in downstream molecular pathways between neurogenic and gliogenic RG mediated by CHRFAM7A. **b** GO analysis of neurogenic oRGs after knockdown of CHRNA7 impacts histone H3-K27 demethylation. **c**. DEGs in oRGs from GW19-22, after knockdown of CHRFAM7A. Knockdown of CHRFAM7A in GW19-23 oRGs led to upregulation of key genes including CACNB2, FBXL7, MAP1B, SLC1A3, NLGN1, TNC, NFIA, PTN, CTNND2 and downregulation of GRIA2, MTSS1 and FBRSL1. SLC1A3, TNC and PTN are known markers of progenitor cells and are highly expressed in oRGs[3]. MAP1B is expressed early in the developing brain and involved in axonal elongation and guidance and neuronal migration[4-8]. CTNND2 interacts with cell-cell junction proteins (cadherins), is involved in cell adhesion and synapses, and has been associated with a variety of neuropsychiatric diseases[9-11]. GRIA2 codes for glutamate AMPA receptor 2, gating an ion channel that mediates fast excitatory synaptic transmission[12]. Other associated GO processes after CHRFAM7A knockdown include oligodendrocyte differentiation, stem and glial differentiation, and myelination, processes not observed at GW15 and active at later developmental stages. **d.** GO analysis of oRG after knockdown of CHRFAM7A identifies functions related to neuron projection and glial cell differentiation. All DEGs and GO are provided in the Source Data. **e.** GO analysis of gliogenic immature neurons includes histone H3-K27 demethylation and glycogen regulation apart from pre- and postsynaptic regulation. **f.** GO analysis of gliogenic upper layer neurons shows postsynaptic membrane assembly and BMP signaling as significant processes post knockdown of CHRNA7. **g.** GO analysis of gliogenic upper layer neurons post knockdown of CHRFAM7A shows sterol transport and PDGFR-b pathway as significant processes. All DEGs and GO are provided in the Source Data.

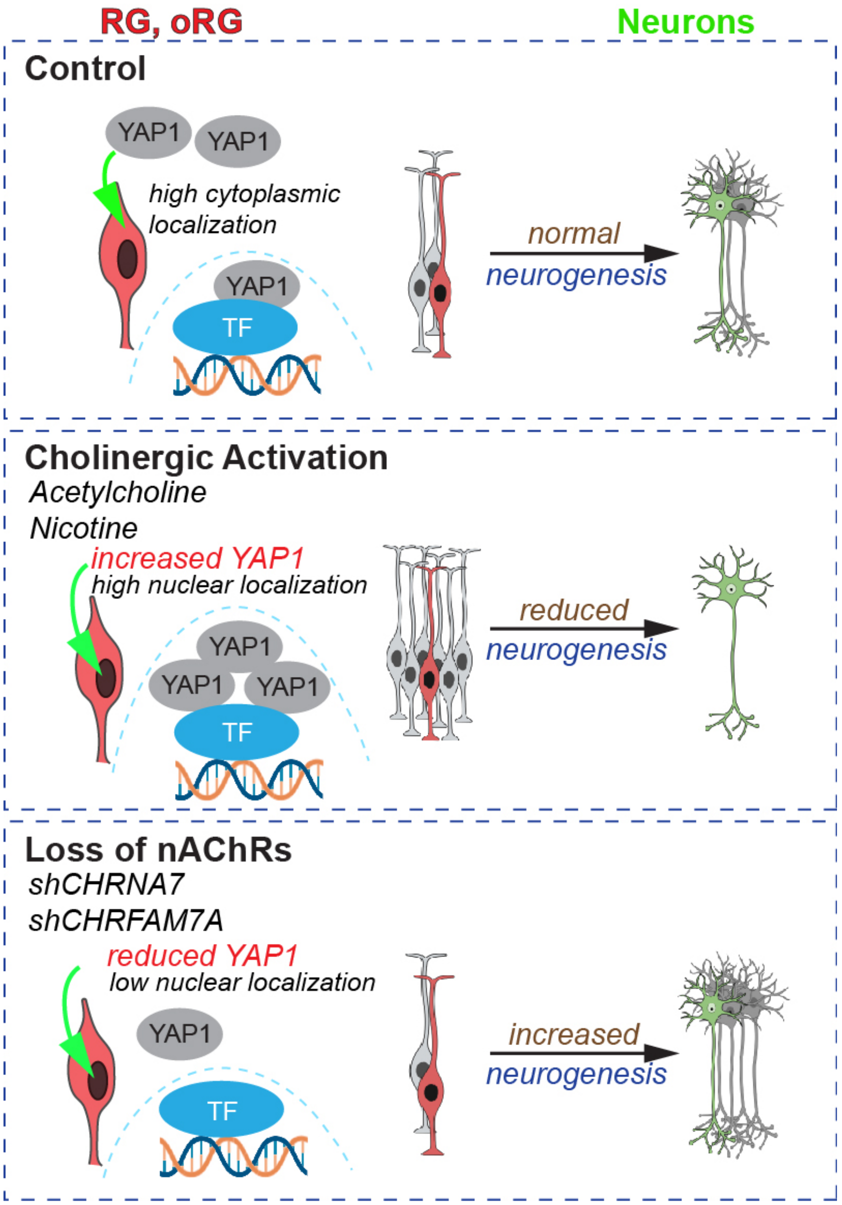


**Supplementary Fig. 10. Working model.** Our working model proposes that nAChRs maintain a tightly regulated balance between stem cell and neurogenic fates (*Created in BioRender. Mukhtar, T. (2025) https://BioRender.com/85db78p*)[97]. Agonist mediated-cholinergic activation causes a shift from neuronal differentiation towards stem cell maintenance and proliferation. The opposite is observed with knockdown of nAChRs; CHRNA7 and CHRFAM7A. These effects are partially mediated by expression and nuclear translocation of the Hippo signaling effector, YAP1. These findings highlight the essential yet underexplored role of cholinergic signaling in modulating Hippo pathway activity, influencing the fate of RG, and oRG, with potential implications for cortical development and disease.
