# Supplementary material for: α7 nicotinic acetylcholine receptors regulate radial glia fate in the developing human cortex
Source: Nat Commun. 2025 Jul 1;16:5925. doi: 10.1038/s41467-025-61167-5 (PMC12217762; doi:10.1038/s41467-025-61167-5)
Supplement: Supplementary file 2 — Reporting Summary [file 41467_2025_61167_MOESM2_ESM.pdf]

nature portfolio

Corresponding author(s): Tanzila Mukhtar and Arnold Kriegstein

Last updated by author(s): 2025/13/05

# Reporting Summary

Nature Portfolio wishes to improve the reproducibility of the work that we publish. This form provides structure for consistency and transparency in reporting. For further information on Nature Portfolio policies, see our Editorial Policies and the Editorial Policy Checklist.

## Statistics

For all statistical analyses, confirm that the following items are present in the figure legend, table legend, main text, or Methods section.

| n/a | Confirmed | |
|---|---|---|
| ☐ | ☒ | The exact sample size (*n*) for each experimental group/condition, given as a discrete number and unit of measurement |
| ☐ | ☒ | A statement on whether measurements were taken from distinct samples or whether the same sample was measured repeatedly |
| ☐ | ☒ | The statistical test(s) used AND whether they are one- or two-sided<br>*Only common tests should be described solely by name; describe more complex techniques in the Methods section.* |
| ☐ | ☒ | A description of all covariates tested |
| ☐ | ☒ | A description of any assumptions or corrections, such as tests of normality and adjustment for multiple comparisons |
| ☐ | ☒ | A full description of the statistical parameters including central tendency (e.g. means) or other basic estimates (e.g. regression coefficient) AND variation (e.g. standard deviation) or associated estimates of uncertainty (e.g. confidence intervals) |
| ☐ | ☒ | For null hypothesis testing, the test statistic (e.g. *F*, *t*, *r*) with confidence intervals, effect sizes, degrees of freedom and *P* value noted<br>*Give P values as exact values whenever suitable.* |
| ☒ | ☐ | For Bayesian analysis, information on the choice of priors and Markov chain Monte Carlo settings |
| ☒ | ☐ | For hierarchical and complex designs, identification of the appropriate level for tests and full reporting of outcomes |
| ☒ | ☐ | Estimates of effect sizes (e.g. Cohen's *d*, Pearson's *r*), indicating how they were calculated |

*Our web collection on statistics for biologists contains articles on many of the points above.*

## Software and code

Policy information about availability of computer code

| | |
|---|---|
| Data collection | No software used |

| | |
|---|---|
| Data analysis | Bioinformatics Analysis- Bulk-RNAseq<br>Reads were aligned and quantified using kallisto and quantified at gene level with tximport. Differential gene expression analysis was performed using limma/voom, accounting for n=2 biological replicates per timepoint, with N=2 technical replicates within each individual. P values were corrected for multiple comparisons using the Benjamini-Hochberg false-discovery rate adjustment (Extended Datasheet 2).<br><br>Bioinformatics Analysis - scRNAseq<br>Nova-Seq data from 8 sublibraries were mapped to GRCh38/hg38 and demultiplexed using split-pipe109 (v1.1.1) from the parse biosciences pipeline with the following parameters: --mode all --chemistry v2 --kit WT. Then the outputs of each sublibrary were combined into a single dataset using split-pipe --mode comb. Gene expression matrices were loaded into the Python package Scanpy110 (1.9.3) for quality control and downstream analyses. We removed cells based on these criteria: (1) number of UMIs ≤ 200, (2) number of UMIs ≥ 98-percentile, and (3) percentage of mitochondrial genes ≥ 20%. Cell doublets were detected and removed using scvi-tools (0.20.3)111. Additionally, genes expressed in less than 10 cells were discarded. Clean data were then normalized and transformed with Scanpy. Then we leveraged scvi-tools to correct batch effects. To cluster the cells and identify cell types, we used Scanpy for downstream analysis.<br>For differential genes analysis, FindMarkers from Seurat112 (v4.3.0) was applied to compare two conditions. We combined GW15 and GW16 as 2nd_early time point, and GW19 and GW22 as 2nd_late time point. Genes with \|log2FoldChange\| > 0.25 and p.adjust <0.05 were considered as differentially expressed genes. For GO terms enrichment, enrichR113 (v3.2) and databases "GO_Molecular_Function_2021", "GO_Cellular_Component_2021", "GO_Biological_Process_2021" were used for functional enrichment. |

For manuscripts utilizing custom algorithms or software that are central to the research but not yet described in published literature, software must be made available to editors and reviewers. We strongly encourage code deposition in a community repository (e.g. GitHub). See the Nature Portfolio guidelines for submitting code & software for further information.

## Data

Policy information about availability of data

All manuscripts must include a data availability statement. This statement should provide the following information, where applicable:

- Accession codes, unique identifiers, or web links for publicly available datasets
- A description of any restrictions on data availability
- For clinical datasets or third party data, please ensure that the statement adheres to our policy

Original data from the study is available in Extended Datasheets 1, 2 and 3. RNA-seq data will be available in dbGAP upon publication at dbGAP study accession: phs000989.v7.p1.

## Research involving human participants, their data, or biological material

Policy information about studies with human participants or human data. See also policy information about sex, gender (identity/presentation), and sexual orientation and race, ethnicity and racism.

| | |
|---|---|
| Reporting on sex and gender | Cell lines with sex information provided. |
| Reporting on race, ethnicity, or other socially relevant groupings | *Please specify the socially constructed or socially relevant categorization variable(s) used in your manuscript and explain why they were used. Please note that such variables should not be used as proxies for other socially constructed/relevant variables (for example, race or ethnicity should not be used as a proxy for socioeconomic status).*<br>*Provide clear definitions of the relevant terms used, how they were provided (by the participants/respondents, the researchers, or third parties), and the method(s) used to classify people into the different categories (e.g. self-report, census or administrative data, social media data, etc.)*<br>*Please provide details about how you controlled for confounding variables in your analyses.* |
| Population characteristics | *Describe the covariate-relevant population characteristics of the human research participants (e.g. age, genotypic information, past and current diagnosis and treatment categories). If you filled out the behavioural & social sciences study design questions and have nothing to add here, write "See above."* |
| Recruitment | *Describe how participants were recruited. Outline any potential self-selection bias or other biases that may be present and how these are likely to impact results.* |
| Ethics oversight | *Identify the organization(s) that approved the study protocol.* |

Note that full information on the approval of the study protocol must also be provided in the manuscript.

# Field-specific reporting

Please select the one below that is the best fit for your research. If you are not sure, read the appropriate sections before making your selection.

[x] Life sciences [ ] Behavioural & social sciences [ ] Ecological, evolutionary & environmental sciences

For a reference copy of the document with all sections, see nature.com/documents/nr-reporting-summary-flat.pdf

# Life sciences study design

All studies must disclose on these points even when the disclosure is negative.

| | |
|---|---|
| Sample size | Every experiment has the sample size mentioned in the respective extended datasheets. Details are also mentioned in the text and figure legends. Multiple biological and technical replicates were used. We found consistent results across all test samples. |
| Data exclusions | No data was excluded |
| Replication | Building on our previous work, we used 3 to 4 high-quality prenatal human cortical samples per experiment alongside 3 iPSC-derived cortical organoids. This sample size reflects the practical constraints of working with rare human tissue and is sufficient to ensure experimental rigor and reproducibility.<br><br>Quantifications were conducted manually using IMARIS Software across several biological replicates. For statistical analysis, an unpaired two-tailed student's t-test using prism software was used. For phenotypic analyses, to handle repeated measures and paired samples when analyzing staining data, a linear mixed effect model [lme4 lmerTest references] was used to statistically model the effect of agonist, antagonist, and shRNA knockdowns. Staining was calculated as a percentage of DAPI cells within the image, which generated an approximately normal distribution of values. Each staining was modeled as follows:<br>Percentage Positive ~ Condition + (1\|Sample).<br>For a given protein stain, models were fit within each tissue type (Organoids week 4, Organoids Week 8, Slice cultures), with only relevant conditions. Untreated results was the background for results from ACh, nicotine, and BTX treatments, DMSO for PNU, and shCONTROL for shChRNA7 and shCHRFAM7A.<br>lme4: https://www.jstatsoft.org/article/view/v067i01114<br>lmerTest: https://www.jstatsoft.org/article/view/v082i13115<br>dharma: https://cran.r-project.org/web/packages/DHARMa/vignettes/DHARMa.html116<br>TUNEL staining was not normally distributed, because cell death was low, it generated more empty values than expected by normal distribution. So instead, we took the mean for each sample/condition, and conducted a paired, 2-tailed t test. |
| Randomization | N/A, We utilized 3 to 4 high-quality prenatal human cortical samples per experiment. Given the scarcity of such rare tissue suitable for organotypic slice preparation, implementing a blinding protocol was not feasible without compromising the integrity or viability of the samples. |
| Blinding | same as above. |

# Reporting for specific materials, systems and methods

We require information from authors about some types of materials, experimental systems and methods used in many studies. Here, indicate whether each material, system or method listed is relevant to your study. If you are not sure if a list item applies to your research, read the appropriate section before selecting a response.

## Materials & experimental systems

| n/a | Involved in the study | |
|---|---|---|
| ☐ | ☒ | Antibodies |
| ☐ | ☒ | Eukaryotic cell lines |
| ☐ | ☐ | Palaeontology and archaeology |
| ☐ | ☐ | Animals and other organisms |
| ☐ | ☐ | Clinical data |
| ☐ | ☐ | Dual use research of concern |
| ☐ | ☐ | Plants |

## Methods

| n/a | Involved in the study | |
|---|---|---|
| ☐ | ☐ | ChIP-seq |
| ☐ | ☐ | Flow cytometry |
| ☐ | ☐ | MRI-based neuroimaging |

## Antibodies

Antibodies used

Target Protein Host species Manufacturer Identifier Dilution
AChE mouse Abcam ab2803 1:500
CHRFAM7A rabbit Aviva system biology OAAN02465 1:250
CTIP2 rat Abcam ab18465 1:500
EOMES sheep R&D AF6166 1:200
HOPX rabbit Proteintech 11419-1-AP 1:250
KI67 rat Abcam ab156956 1:300
NEUN guinea pig Millipore ABN90 1:500
SOX2 mouse Santa Cruz Sc-365823 1:500
SATB2 mouse Abcam ab51502 1:500
VIMENTIN chicken EMD Millipore Ab5733 1:250
ZO-1 rabbit Proteintech 21773-1-AP 1:300

DLX5 rabbit Sigma HPA005670 1:100
YAP1 rabbit Novus Biologicals NB110-58358SS 1:300
DCHS1 rabbit Novus Biologicals NBP2-13901 1:300
Primary GAPDH mouse Sigma G8975 1:5,000
Primary HISTONE H3 mouse Cell Signaling 3638S 1:1000
Primary B-ACTIN rabbit Cell Signaling 4970S 1:1000
Primary CHRNA7 rabbit Alomone labs ANC-007 1:500
Primary CHRFAM7A rabbit Aviva system biology OAAN02465 1:500
Primary CHRNB1 rabbit Proteintech 11553-1-AP 1:500
Primary CHRNB2 rabbit Abcam ab41174 1:500
Secondary Anti-Mouse HRP rabbit Abcam ab6728 1:10,000
Secondary Anti-Rabbit HRP goat Abcam ab6721 1:10,000

Validation: Commercially available antibodies were used

## Eukaryotic cell lines

Policy information about cell lines and Sex and Gender in Research

Cell line source(s): Cell line (Homo sapiens) H1 / WA01
Embryonic stem cell line WiCell RRID:CVCL_9771 Male
Cell line (Homo sapiens) WTC-11 induced pluripotent stem cell line Conklin Laboratory (Gladstone Institutes) GM25256 Male
Cell line
(Homo sapiens) 1323-4 induced pluripotent stem cell line Conklin lab (Gladstone Institute) RRID:CVCL_0G84 Female

Authentication: Lab stocks used. Tested for any genetic abnormalities using Karyotyping regularly.

Mycoplasma contamination: Negative

Commonly misidentified lines (See ICLAC register): *Name any commonly misidentified cell lines used in the study and provide a rationale for their use.*

## Palaeontology and Archaeology

Specimen provenance: *Provide provenance information for specimens and describe permits that were obtained for the work (including the name of the issuing authority, the date of issue, and any identifying information). Permits should encompass collection and, where applicable, export.*

Specimen deposition: *Indicate where the specimens have been deposited to permit free access by other researchers.*

Dating methods: *If new dates are provided, describe how they were obtained (e.g. collection, storage, sample pretreatment and measurement), where they were obtained (i.e. lab name), the calibration program and the protocol for quality assurance OR state that no new dates are provided.*

☐ Tick this box to confirm that the raw and calibrated dates are available in the paper or in Supplementary Information.

Ethics oversight: *Identify the organization(s) that approved or provided guidance on the study protocol, OR state that no ethical approval or guidance was required and explain why not.*

Note that full information on the approval of the study protocol must also be provided in the manuscript.

## Animals and other research organisms

Policy information about studies involving animals; ARRIVE guidelines recommended for reporting animal research, and Sex and Gender in Research

Laboratory animals: *For laboratory animals, report species, strain and age OR state that the study did not involve laboratory animals.*

Wild animals: *Provide details on animals observed in or captured in the field; report species and age where possible. Describe how animals were caught and transported and what happened to captive animals after the study (if killed, explain why and describe method; if released, say where and when) OR state that the study did not involve wild animals.*

Reporting on sex: *Indicate if findings apply to only one sex; describe whether sex was considered in study design, methods used for assigning sex. Provide data disaggregated for sex where this information has been collected in the source data as appropriate; provide overall numbers in this Reporting Summary. Please state if this information has not been collected. Report sex-based analyses where performed, justify*

| | |
|---|---|
| | *reasons for lack of sex-based analysis.* |
| Field-collected samples | *For laboratory work with field-collected samples, describe all relevant parameters such as housing, maintenance, temperature, photoperiod and end-of-experiment protocol OR state that the study did not involve samples collected from the field.* |
| Ethics oversight | *Identify the organization(s) that approved or provided guidance on the study protocol, OR state that no ethical approval or guidance was required and explain why not.* |

Note that full information on the approval of the study protocol must also be provided in the manuscript.

# Clinical data

Policy information about clinical studies

All manuscripts should comply with the ICMJEguidelines for publication of clinical research and a completedCONSORT checklist must be included with all submissions.

| | |
|---|---|
| Clinical trial registration | *Provide the trial registration number from ClinicalTrials.gov or an equivalent agency.* |
| Study protocol | *Note where the full trial protocol can be accessed OR if not available, explain why.* |
| Data collection | *Describe the settings and locales of data collection, noting the time periods of recruitment and data collection.* |
| Outcomes | *Describe how you pre-defined primary and secondary outcome measures and how you assessed these measures.* |

# Dual use research of concern

Policy information about dual use research of concern

## Hazards

Could the accidental, deliberate or reckless misuse of agents or technologies generated in the work, or the application of information presented in the manuscript, pose a threat to:

| No | Yes | |
|---|---|---|
| ☐ | ☐ | Public health |
| ☐ | ☐ | National security |
| ☐ | ☐ | Crops and/or livestock |
| ☐ | ☐ | Ecosystems |
| ☐ | ☐ | Any other significant area |

## Experiments of concern

Does the work involve any of these experiments of concern:

| No | Yes | |
|---|---|---|
| ☐ | ☐ | Demonstrate how to render a vaccine ineffective |
| ☐ | ☐ | Confer resistance to therapeutically useful antibiotics or antiviral agents |
| ☐ | ☐ | Enhance the virulence of a pathogen or render a nonpathogen virulent |
| ☐ | ☐ | Increase transmissibility of a pathogen |
| ☐ | ☐ | Alter the host range of a pathogen |
| ☐ | ☐ | Enable evasion of diagnostic/detection modalities |
| ☐ | ☐ | Enable the weaponization of a biological agent or toxin |
| ☐ | ☐ | Any other potentially harmful combination of experiments and agents |

# Plants

Seed stocks

*Report on the source of all seed stocks or other plant material used. If applicable, state the seed stock centre and catalogue number. If plant specimens were collected from the field, describe the collection location, date and sampling procedures.*

Novel plant genotypes

*Describe the methods by which all novel plant genotypes were produced. This includes those generated by transgenic approaches, gene editing, chemical/radiation-based mutagenesis and hybridization. For transgenic lines, describe the transformation method, the number of independent lines analyzed and the generation upon which experiments were performed. For gene-edited lines, describe the editor used, the endogenous sequence targeted for editing, the targeting guide RNA sequence (if applicable) and how the editor was applied.*

Authentication

*Describe any authentication procedures for each seed stock used or novel genotype generated. Describe any experiments used to assess the effect of a mutation and, where applicable, how potential secondary effects (e.g. second site T-DNA insertions, mosiacism, off-target gene editing) were examined.*

# ChIP-seq

## Data deposition

☐ Confirm that both raw and final processed data have been deposited in a public database such as GEO.

☐ Confirm that you have deposited or provided access to graph files (e.g. BED files) for the called peaks.

Data access links
*May remain private before publication.*

*For "Initial submission" or "Revised version" documents, provide reviewer access links. For your "Final submission" document, provide a link to the deposited data.*

Files in database submission

*Provide a list of all files available in the database submission.*

Genome browser session
(e.g. UCSC)

*Provide a link to an anonymized genome browser session for "Initial submission" and "Revised version" documents only, to enable peer review. Write "no longer applicable" for "Final submission" documents.*

## Methodology

Replicates

*Describe the experimental replicates, specifying number, type and replicate agreement.*

Sequencing depth

*Describe the sequencing depth for each experiment, providing the total number of reads, uniquely mapped reads, length of reads and whether they were paired- or single-end.*

Antibodies

*Describe the antibodies used for the ChIP-seq experiments; as applicable, provide supplier name, catalog number, clone name, and lot number.*

Peak calling parameters

*Specify the command line program and parameters used for read mapping and peak calling, including the ChIP, control and index files used.*

Data quality

*Describe the methods used to ensure data quality in full detail, including how many peaks are at FDR 5% and above 5-fold enrichment.*

Software

*Describe the software used to collect and analyze the ChIP-seq data. For custom code that has been deposited into a community repository, provide accession details.*

# Flow Cytometry

## Plots

Confirm that:

☐ The axis labels state the marker and fluorochrome used (e.g. CD4-FITC).

☐ The axis scales are clearly visible. Include numbers along axes only for bottom left plot of group (a 'group' is an analysis of identical markers).

☐ All plots are contour plots with outliers or pseudocolor plots.

☐ A numerical value for number of cells or percentage (with statistics) is provided.

## Methodology

Sample preparation

*Describe the sample preparation, detailing the biological source of the cells and any tissue processing steps used.*

Instrument

*Identify the instrument used for data collection, specifying make and model number.*

Software

*Describe the software used to collect and analyze the flow cytometry data. For custom code that has been deposited into a community repository, provide accession details.*

Cell population abundance

*Describe the abundance of the relevant cell populations within post-sort fractions, providing details on the purity of the samples and how it was determined.*

Gating strategy

*Describe the gating strategy used for all relevant experiments, specifying the preliminary FSC/SSC gates of the starting cell population, indicating where boundaries between "positive" and "negative" staining cell populations are defined.*

☐ Tick this box to confirm that a figure exemplifying the gating strategy is provided in the Supplementary Information.

# Magnetic resonance imaging

## Experimental design

Design type

*Indicate task or resting state; event-related or block design.*

Design specifications

*Specify the number of blocks, trials or experimental units per session and/or subject, and specify the length of each trial or block (if trials are blocked) and interval between trials.*

Behavioral performance measures

*State number and/or type of variables recorded (e.g. correct button press, response time) and what statistics were used to establish that the subjects were performing the task as expected (e.g. mean, range, and/or standard deviation across subjects).*

## Acquisition

Imaging type(s)

*Specify: functional, structural, diffusion, perfusion.*

Field strength

*Specify in Tesla*

Sequence & imaging parameters

*Specify the pulse sequence type (gradient echo, spin echo, etc.), imaging type (EPI, spiral, etc.), field of view, matrix size, slice thickness, orientation and TE/TR/flip angle.*

Area of acquisition

*State whether a whole brain scan was used OR define the area of acquisition, describing how the region was determined.*

Diffusion MRI ☐ Used ☐ Not used

## Preprocessing

Preprocessing software

*Provide detail on software version and revision number and on specific parameters (model/functions, brain extraction, segmentation, smoothing kernel size, etc.).*

Normalization

*If data were normalized/standardized, describe the approach(es): specify linear or non-linear and define image types used for transformation OR indicate that data were not normalized and explain rationale for lack of normalization.*

Normalization template

*Describe the template used for normalization/transformation, specifying subject space or group standardized space (e.g. original Talairach, MNI305, ICBM152) OR indicate that the data were not normalized.*

Noise and artifact removal

*Describe your procedure(s) for artifact and structured noise removal, specifying motion parameters, tissue signals and physiological signals (heart rate, respiration).*

Volume censoring

*Define your software and/or method and criteria for volume censoring, and state the extent of such censoring.*

## Statistical modeling & inference

Model type and settings

*Specify type (mass univariate, multivariate, RSA, predictive, etc.) and describe essential details of the model at the first and second levels (e.g. fixed, random or mixed effects; drift or auto-correlation).*

Effect(s) tested

*Define precise effect in terms of the task or stimulus conditions instead of psychological concepts and indicate whether ANOVA or factorial designs were used.*

Specify type of analysis: ☐ Whole brain ☐ ROI-based ☐ Both

Statistic type for inference

(See Eklund et al. 2016)

*Specify voxel-wise or cluster-wise and report all relevant parameters for cluster-wise methods.*

Correction

*Describe the type of correction and how it is obtained for multiple comparisons (e.g. FWE, FDR, permutation or Monte Carlo).*

## Models & analysis

| n/a | Involved in the study |
| --- | --- |
| ☐ | ☐ Functional and/or effective connectivity |
| ☐ | ☐ Graph analysis |
| ☐ | ☐ Multivariate modeling or predictive analysis |

| | |
| --- | --- |
| Functional and/or effective connectivity | *Report the measures of dependence used and the model details (e.g. Pearson correlation, partial correlation, mutual information).* |
| Graph analysis | *Report the dependent variable and connectivity measure, specifying weighted graph or binarized graph, subject- or group-level, and the global and/or node summaries used (e.g. clustering coefficient, efficiency, etc.).* |
| Multivariate modeling and predictive analysis | *Specify independent variables, features extraction and dimension reduction, model, training and evaluation metrics.* |
